# Differences in Outcomes Based on the Degree to Which Bone Defects Are Filled with Cancellous Allochip Bone Grafts in Hand Enchondroma Patients

**DOI:** 10.3390/cancers16223811

**Published:** 2024-11-13

**Authors:** Sung Ju Kang, Jun-Hyuk Lim, Chan Wi Kim, Gyo Rim Kang, Sungmin Kim, Sung-Taek Jung

**Affiliations:** 1 Department of Orthopedic Surgery, Chonnam National University Medical School and Hospital, 42 Jebongro, Donggu, Gwangju 61469, Republic of Korea; ksjlv5955@naver.com (S.J.K.); ove03@naver.com (J.-H.L.); ckdnl77@naver.com (C.W.K.); 2 Department of Orthopedic Surgery, Chonnam National University Hospital, Donggu, Gwangju 61469, Republic of Korea; gyorim0120@gmail.com

**Keywords:** enchondroma, curettage, impaction grafting, bone defect filling, outcomes

## Abstract

Background/Objectives: Enchondroma, the most common benign cartilage tumor in the hand, often presents as pain, swelling, or pathological fractures. While curettage is the standard treatment preventing fractures, the optimal way of managing the bone cavity remains debated. In this study, we investigated the impact of a filled bone defect on radiologic and clinical outcomes among patients with enchondroma. Methods: We retrospectively reviewed patients with solitary enchondroma of the hand who underwent curettage followed by allogeneic cancellous bone chip impaction grafting. The patients were divided into two groups based on the extent to which their bone defects were filled post-curettage: Group 1 (complete filling) and Group 2 (incomplete filling, i.e., <50%). We reviewed demographic data, local recurrence data, complications, information on consolidation duration, data on range of motion (ROM), and functional scores. Results: This study included 59 patients (25 males and 34 females; mean age, 30.4 ± 11.9 years, with a range of 8–78). Group 1 contained 35 patients, and Group 2 contained 24. No nonunion occurred following curettage and grafting. The mean radiological consolidation period was 6.4 weeks (range: 5–18). There was no significant difference in consolidation time between Group 1 (6.8 weeks) and Group 2 (6.9 weeks) (*p* = 0.166). The ROM and functional scores also showed no significant differences between the groups, with musculoskeletal society scores of 98.8 for Group 1 and 99.8 for Group 2 (*p* = 0.63). Conclusions: This study demonstrates that the use of the impaction technique combined with cancellous allochip bone grafting yields favorable results in the treatment of solitary hand enchondroma. The extent to which the bone defect was filled did not significantly impact the overall outcomes.

## Simple Summary

Enchondromas are benign tumors that commonly affect the hand and can cause pain, swelling, and fractures. Standard treatment involves scraping out the tumor and filling the resulting cavity with bone grafts. This study examined how thoroughly filling this cavity affects outcomes after this procedure. We reviewed 59 patients who had their enchondromas treated via bone grafting and divided them into two groups: those whose cavities were completely filled with grafts (Group 1) and those with less than half-filled cavities (Group 2). We examined their recovery times, ranges of motion, and functional scores. The results show no significant differences between the two groups. Both groups had similar recovery times, range of motion, and functional outcomes, suggesting that overall treatment success is not greatly impacted by whether the cavity is fully or partially filled.

## 1. Introduction

Enchondroma is the most common benign hyaline cartilage tumor that occurs within the medullary cavity of the hand bones, accounting for over 50% of cases in this location [1,2,3,4,5]. It is often diagnosed incidentally via radiographs or due to symptoms such as local pain, swelling, or pathological fractures. Radiographs typically reveal a well-defined lytic lesion, which may or may not show calcification, and usually does not affect the bone cortex or adjacent soft tissues [6,7,8].

Although enchondromas are relatively slow-growing tumors, they can weaken bone structure, leading to pathological fractures, a common complication. These tumors typically present as solitary lesions in the phalanges. The primary goals of surgical treatment are to confirm the diagnosis through histopathological examination following curettage and prevent future pathological fractures by stabilizing the bone [9,10].

While there is a consensus on using curettage as the surgical approach for enchondroma, debate continues regarding the optimal management of the resulting bone cavity. Typically, the cavity is filled with either biological or synthetic materials [11,12]. On the other hand, the extent to which the bone defect is filled remains a topic of ongoing discussion, with some recent studies suggesting that even simple curettage or incomplete filling may lead to favorable clinical and radiologic outcomes, challenging the necessity of complete defect filling in all cases [4,13].

We aimed to investigate whether the extent of bone defect filling influences radiologic and clinical outcomes among patients with enchondroma.

## 2. Materials and Methods

**Figure 1:**
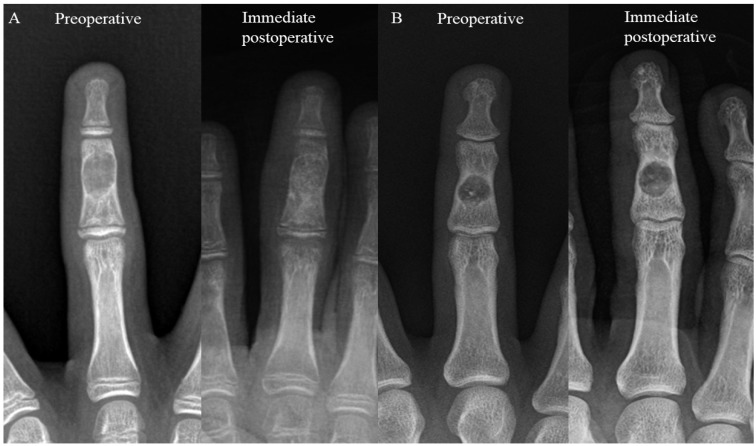
This is an enchondroma of the middle phalanx of the lt 3rd finger treated via curettage and allogenic cancellous bone chip impaction grafting. (**A**) A 14-year-old male patient who underwent curettage and complete filling, categorized as Group 1. (**B**) A 28-year-old male patient who underwent curettage and had less than 50% (i.e., incomplete) of the affected area filled, categorized as Group 2.

In this retrospective study, medical records were reviewed to identify patients with histologically confirmed solitary enchondroma of the hand who underwent simple curettage followed by impaction grafting with allogenic cancellous bone chips between January 2010 and July 2020. The inclusion criteria were (1) histologically confirmed solitary enchondroma, (2) having undergone simple curettage followed by impaction grafting using allogenic cancellous bone chips, and (3) a minimum postoperative follow-up of one year. The exclusion criteria included (1) patients with multiple lesions, (2) those diagnosed with malignancies such as low-grade or high-grade chondrosarcoma, and (3) patients lost to follow-up. The patients were categorized into two groups based on the extent of bone defect filling post curettage identified in radiographs. Group 1 included those with complete filling, defined as more than 90% of the cavity being filled, while Group 2 included those with incomplete filling, defined as less than 50% of the cavity being filled (Figure 1) [14].

### 2.1. Surgical Procedure and Rehabilitation

After confirming the lesion using fluoroscopy, a burr was used to create a window in the cortical bone. Curettage was then performed until normal cancellous bone and cortical bone were observable. Following adequate irrigation, allogenic cancellous bone chips were impacted into the defect [15]. Whenever possible, we aimed to completely fill the bone defect using allogenic bone chips.

Postoperatively, a short arm splint was applied to immobilize the hand, and it remained in place for approximately 6 weeks. For the first 2 weeks, the hand was kept immobilized without asking the patient to perform any range of motion (ROM) exercises. Starting in the third week, the patient was instructed to perform gentle, passive ROM exercises once a day. The patients were permitted to gradually begin performing active ROM exercises starting one month post surgery.

### 2.2. Radiographic Evaluation

The patients attended their first outpatient follow-ups 2 weeks after the operation, followed by subsequent visits every 4 weeks from 1 to 3 months postoperatively. From 3 months to 1 year post operation, follow-up appointments were scheduled every 3 months. After 1 year, the patients were seen annually.

Preoperatively, anteroposterior (AP), lateral, and oblique plain radiographs of the affected hand were obtained. Postoperatively, during the follow-up period, these same radiographs were taken.

The radiographic size of the enchondroma was defined as the largest dimension in the AP or lateral plain radiograph view of the affected extremity in centimeters [16].

Radiographic consolidation (healing) was defined as the presence of normal cortical bone in plain radiographs post surgery, with the bone defect being less than 3 mm [15].

Recurrence was defined by the identification of new lucent areas upon conducting postoperative imaging that were not present in intraoperative or immediate postoperative films [15].

### 2.3. Clinical Evaluation

Age, sex, the duration of symptoms, recurrence, fractures, and the recurrence of symptoms were reviewed. ROM was assessed using the Total Active Motion (TAM) of the affected finger both preoperatively and at 6 months postoperatively. TAM is defined as the sum of the active range of motion in flexion and extension across the metacarpophalangeal (MCP), proximal interphalangeal (PIP), and distal interphalangeal (DIP) joints, measured in degrees. For reference, normal TAM values are reported as 260 degrees for fingers and 140 degrees for the thumb. Additionally, the Musculoskeletal Tumor Society (MSTS) score [17], which evaluates functional outcomes, was also assessed one year post operation.

### 2.4. Statistical Analysis

A chi-square test was used for the statistical analysis of the demographic data. One-way ANOVA was employed to compare the differences between groups in terms of the follow-up outcomes (SPSS ver. 19.0). Data were expressed as mean ± standard deviations. Statistical significance was considered at the *p* ˂ 0.05 level.

## 3. Results

A total of 59 patients were included in this study, comprising 25 males and 34 females, with an average age of 30.4 ± 11.9 (range, 8–78). All lesions were histologically diagnosed as enchondromas. The mean follow-up duration post surgery was 28.8 ± 12.9 months (range, 12–67 months).

The middle finger was the most affected, accounting for 18 cases (30.5%), followed by the little finger with 13 cases (22.0%), the ring finger and index finger, each with 12 cases (20.3%), and the thumb, which was affected in 4 cases. The proximal phalanx was the most frequently involved site, observed in 24 cases (40.6%), followed by the middle phalanx in 14 cases (23.7%), the distal phalanx in 10 cases (16.9%), and the metacarpal bones in 11 cases (18.6%).

Thirty-five and twenty-four patients were categorized into Groups 1 and 2, respectively. No patients experienced nonunion following curettage and allogenic bone grafting. The mean radiological consolidation period was 6.4 weeks (range, 5–18 weeks). The consolidation times between the two groups were not significantly different, with Group 1 at 6.8 weeks (range, 5–16 weeks) and Group 2 at 6.9 months (range, 6–18 months) (*p* = 0.166).

The TAM values obtained preoperatively and at 6 months post surgery are detailed in Table 1. No significant difference in ROM was observed based on the extent of cavity filling. Additionally, there was no significant difference in MSTS scores between the two groups (*p* = 0.63). The MSTS scores were 98.8 (range, 93.3–100) for Group 1 and 99.8 (range, 96.7–100) for Group 2.

**Figure 2:**
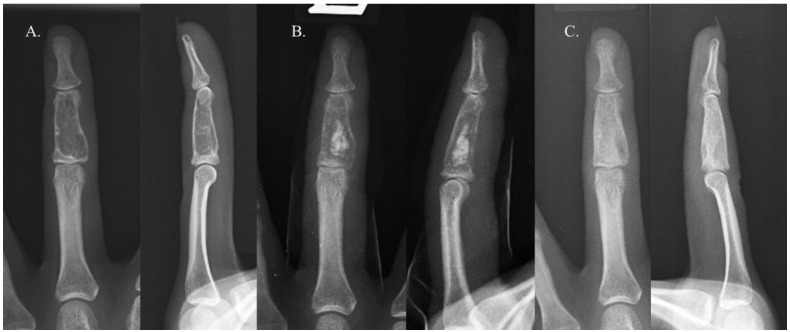
A 22-year-old female with an enchondroma in the middle phalanx of her left third finger. The images show simple radiographs taken before surgery (**A**), immediately after surgery (**B**), and 1 year after surgery (**C**). The bone graft filled approximately 39% of the defect, categorizing it as Group B.

**Figure 3:**
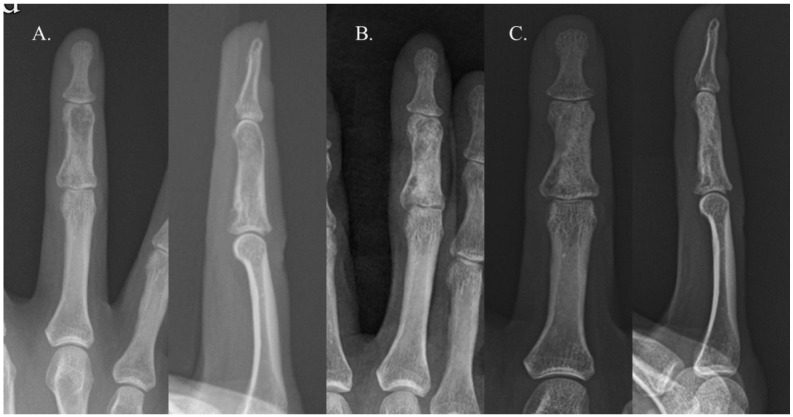
Simple radiographs of a patient with recurrence 6 years (**A**), immediately after surgery (**B**), and 1 year after surgery (**C**).

Recurrence occurred in only one case. The patient in question was a 22-year-old woman with an enchondroma in the middle phalanx of her left third finger. She underwent curettage and allogenic cancellous chip bone grafting. The bone graft filled approximately 39% of the defect, categorizing it as Group B (Figure 2). Sixteen years later, the enchondroma recurred in the same location. Repeated curettage and allogenic cancellous chip bone grafting were performed, and pathological analysis confirmed the presence of an enchondroma (Figure 3). There were no complications, such as infection, malunion, persistent pain, or refracture, in either group.

## 4. Discussion

Whether curettage alone is sufficient or if a bone defect should be filled to some extent when treating solitary enchondroma of the hand remains a subject of ongoing debate. In our study, we divided patients with a solitary hand enchondroma based on the degree to which it was filled with allogenic bone graft material after curettage and compared the clinical and radiological outcomes accordingly.

We observed no significant differences in postoperative clinical scores, ROM, or consolidation duration among patients, regardless of the degree of cavity filling. These findings align with the existing literature on the impact of grafting and cavity filling following curettage. Sassoon et al. [18] reported that using allografts or leaving the cavity unfilled was advantageous due to the lack of donor site morbidity, with no negative impact on healing time, recurrence, or functional outcomes. Similarly, studies by Morii et al. [19] and Schaller et al. [20] emphasized that simple curettage without augmentation is a safe and effective treatment for small enchondromas, particularly in the hand and foot. They suggest that additional bone grafting should be reserved for specific cases, a conclusion that is consistent with our findings. Overall, our results indicate that the necessity of filling the bone defect after curettage may be less critical than previously assumed.

There is still considerable debate regarding which material should be used to fill the bone defect after curettage. However, several studies have reported that the type of material used does not significantly influence the outcomes [3,18,21,22]. Autologous bone grafts have the advantage of promoting the fastest consolidation [15,21], but they carry the risk of donor site morbidity and are limited by the available harvest volume. Additionally, some studies have reported a higher incidence of complications when autologous bone grafts are used [23,24,25,26]. For these reasons, the use of synthetic bone substitutes has been on the rise [15]. While synthetic bone substitutes are convenient to use, they also have drawbacks, such as the potential for foreign body reactions and longer consolidation times [27,28]. In our study, we used allogenic cancellous bone chips, and we did not observe any complications such as foreign body reactions, infection, or nonunion, which is consistent with the findings of other studies [11,29].

In our study, the mean consolidation period was 6.4 weeks (range, 5–18 weeks) with no significant difference between the two groups (6.4 weeks vs. 6.9 weeks, *p* = 0.166). Consolidation time after the treatment of hand enchondroma has been reported to vary widely, ranging from 4 weeks to 12 months [15,21,27]. Specifically, when synthetic bone grafts are used, bone integration has been documented to take approximately 9 to 12 months [27]. Interestingly, even when bone grafting is not performed after simple curettage, the consolidation period does not differ significantly. Morii et al. [19] observed 38 patients over an average of 24 months and reported a consolidation period of approximately 6.5 weeks. Bachoura et al. [26] followed up with 26 lesions for an average of 26 months and found that complete consolidation occurred for 68% of patients. In our study, the difference in bone graft filling did not significantly impact the timing of consolidation, a result consistent with these findings. It appears that in the treatment of hand enchondroma, simple curettage plays a decisive role, and the extent to which the bone defect is filled does not significantly affect the healing process. However, there is a possibility that the use of the impaction technique may have contributed to increased structural stability [30]. Allogeneic cancellous bone chip impaction grafting appears to be a safe and efficient surgical technique for treating solitary enchondroma of the hand, regardless of the extent to which the bone defect is filled.

The limitations of this study include its retrospective design, which inherently carries potential biases such as selection bias and recall bias. Additionally, this study’s small sample size limits the generalizability of the findings, as a larger cohort might reveal different trends or outcomes that were not observable in this study. Another significant limitation is the absence of a control group that underwent only curettage without any bone graft filling. This lack of a comparative group makes it difficult to fully assess the independent effects of the bone grafting procedure on the consolidation period and overall clinical outcomes. This study’s findings, therefore, should be interpreted with caution, and further prospective studies with larger sample sizes and appropriate control groups are necessary to validate these results and provide a more comprehensive understanding of the optimal treatment approach for solitary enchondroma of the hand. 

## 5. Conclusions

In conclusion, this study suggests that the use of the impaction technique combined with cancellous allochip bone grafting yields favorable results in the treatment of solitary hand enchondroma, but the degree of cancellous bone graft filling may not significantly influence surgical outcomes for hand enchondromas.

## Figures and Tables

**Table 1:** Total Active Motion (TAM) before the operation and 6 months post surgery.

	Group 1 (*n* = 35)	Group 2 (*n* = 24)	*p*-Value
Preoperative TAM (degree)	198.2 (78–250)	197.6 (79–250)	0.14
TAM at 6 months post surgery (degree)	239.2 (range, 210–250)	238.9 (range, 210–250)	0.16

## Data Availability

Data supporting reported results can be found in patient medical files located in the archive of Chonnam National University Hospital.
